# Histopathological Variants of Cutaneous Squamous Cell Carcinoma: A Review

**DOI:** 10.1155/2011/210813

**Published:** 2010-12-29

**Authors:** Valerie R. Yanofsky, Stephen E. Mercer, Robert G. Phelps

**Affiliations:** ^1^Albert Einstein College of Medicine, Bronx, NY 10461, USA; ^2^Division of Dermatopathology, Mount Sinai School of Medicine, One Gustave L. Levy Place, NY 10029, USA

## Abstract

Nonmelanoma skin cancer (NMSC) is the most common form of cancer in the Caucasian population, with squamous cell carcinoma (SCC) accounting for the majority of NMSC-related metastases and death. While most SCC lesions are indolent tumors with low malignant potential, a wide diversity of SCC subtypes exist, several of which are associated with markedly more aggressive behaviors. Distinguishing these high-risk variants from their counterparts is possible through microscopic analysis, since each subtype possesses unique histopathological features. Early identification of high-risk lesions can allow for more rapid therapeutic intervention, reducing the likelihood of metastasis and death. The authors review specific histopathological features and associated clinical outcomes of the primary subdivisions of SCC.

## 1. Introduction

Nonmelanoma skin cancer (NMSC) is the most common form of cancer seen in the Caucasian population [1]. The term NMSC can theoretically be applied to all cutaneous cancers excluding melanoma, however, it is most commonly used to refer to the two major types of skin cancers: basal cell carcinoma (BCC), and cutaneous squamous cell carcinoma (SCC). Together these forms account for over 95% of all NMSC, with SCC accounting for approximately 20% of all cutaneous malignancies [2, 3]. Despite their lower frequency, SCCs account for the majority of NMSC-related metastatic disease and death, and are reported to be within the top five most costly cancers in the United States (US) [4]. Studies also confirm a dramatic increase in the incidence of SCC over the past several decades [5], which can be attributed to, amongst other things, an increase in sun exposure, intensifying UV exposure, the advancing age of the US population, enhanced public awareness of skin cancer, and more frequent skin examinations by physicians [5]. 

While numerous subtypes of BCC have been described according to their microscopic appearance, most of these variants will demonstrate little significant difference in biological behaviors, with minimal competence for distant spread. In contrast, there exists a wide histopathologic diversity of SCCs, many of which are associated with markedly different clinical behaviors. These can range from indolent tumors with low metastatic potential, to remarkably aggressive tumors with high invasive potential [6–9]. The ability to distinguish between these variants microscopically is thus critically important in the clinical diagnosis and treatment of SCC, with early treatment of high-risk tumors resulting in better patient outcomes with a lower risk of tumor metastasis and recurrence [4, 10].

The purpose of this review is thus two fold. First, we aim to provide a clear and comprehensive means of discriminating between various SCC lesions on the basis of intrinsic differences in their histopathology. This will include a detailed description of the unique histopathological features pertaining to each SCC subtype, highlighting the often subtle differences which can be used in their distinction. More specifically, our descriptive analysis can be loosely organized into three separate categories and will encompass: actinic or solar keratoses (AKs) and SCC *in situ* (Bowen's disease), common precursors to SCC formation seen as a direct result of excess sun exposure; invasive SCC (SCCI), clear-cell SCC, spindle cell (sarcomatoid) SCC, and SCC with single cell infiltrates, tumor subtypes which emerge from the invasive progression of the aforementioned lesions; *de novo* SCC, lymphoepithelioma-like carcinoma of the skin (LELCS), and verrucous carcinoma (VC), highly uncommon SCC variants with no direct correlation to sun exposure or actinic precursors. While there are undoubtedly several other rare subtypes of SCC and squamoid neoplasms which we will not be addressing directly, the vast majority of commonly seen SCCs can be incorporated into one of the categories mentioned above. 

The second aim of this paper is to highlight those variants of SCC with the greatest malignant potential, considered to be high-risk SCCs. This will facilitate the clinician in making a more efficient and better informed selection of treatment options, thus ensuring the most appropriate and effective care for the patient.

## 2. Actinic Keratosis (AK)

AKs are widely accepted as precancerous lesions which act as precursors to SCC formation. They develop as the result of excess UV damage on sun-exposed surfaces of the body, including the face, neck, dorsal hands, and forearms, upper chest, back, and scalp [7, 9, 11, 12]. They are more likely to arise in the Type 1 and Type 2 skin populations, comprised of fair-skinned individuals with a high propensity to burn. Since the incidence of these tumors is directly correlated to sun exposure, they will generally present in middle-aged or older individuals. They can, however, occasionally be found in younger individuals, particularly in those who are more likely to accrue UV damage due to predisposing factors such as immune suppression [12]. Recently, there has been an increasing trend of AKs presenting in younger patients, due in large part to elevated and prolonged levels of sun exposure [13]. 

Clinically, AKs will manifest as ill-marginated, erythematous, scaling, and rough papules or patches. These will typically be found in areas displaying other signs of solar damage, such as atrophy, uneven pigmentation, and telangiectasias [14]. Lesions are further associated with three possible clinical outcomes, which include spontaneous regression, persistence as a benign AK, or evolution into an invasive SCC (SCCI) [15]. Although the overwhelming majority of SCCs are found to be associated with AKs [16], these lesions are generally considered to have a low malignant potential, with only 5%–10% progressing to SCCI over the next several years [17]. 

Histopathologically, several different variants of AKs have been identified, including hypertrophic, atrophic, acantholytic, pigmented, proliferative, and Bowenoid subtypes. In general, the hypertrophic and proliferative variants are associated with more aggressive biologic behaviors and have a higher malignant potential [7, 9, 18, 19]. 

**Figure 1:**
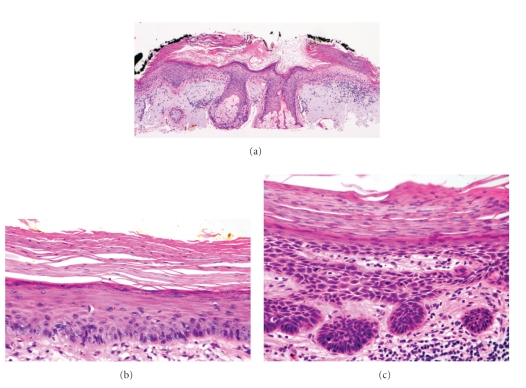
Actinic keratosis (AK). (a) One of the first clues to the diagnosis of AK at scanning magnification is the discontinuity of the parakeratosis as the dysplastic process spares adnexal structures. Note the lack of parakeratosis over the sebaceous gland. This specimen also demonstrates dense dermal elastosis (40x). (b) Example of an early AK with keratinocyte dysplasia confined to the lower third of the epidermis (200x). (c) A more established lesion of AK demonstrating nearly full thickness keratinocyte dysplasia and prominent budding of the basal layer into the superficial dermis (200x).

By definition, AKs are confined to foci within the epidermis. They are associated with aggregates of atypical, pleomorphic keratinocytes which show nuclear atypia, dyskeratosis, and loss of polarity. Hyperkeratosis and parakeratosis are often seen, the latter overlying the abnormal cells in the epidermis. Due to the sparing of segments of the epithelium overlying adnexal structures, a characteristic pattern of alternating orthokeratosis and parakeratosis, referred to as the “flag-sign,” can often be seen (Figure 1(a)) [20]. Atypical keratinocytes from the basal cell layer will frequently extend into the granular and cornified layers, however, they will not span the full thickness of the epidermis (Figure 1(b)) [20, 21]. The exception to this criterion is the Bowenoid variant of AK, which resembles Bowen's disease but is less disordered with less nuclear atypia and crowding [9]. The basal layer in AKs will often appear to be more basophilic than normal, which is generally thought to be a consequence of the close crowding of atypical keratinocytes (Figure 1(b)). Some cases will also show basal layer degeneration and the formation of Civatte bodies, the result of a lichenoid infiltrate with irregular acanthosis. This can be distinguished from lichenoid dermatitis by the presence of keratinocyte atypia [22]. 

The dermoepidermal junction in AKs will also show irregularities, with small round buds at the basal cell layer that will protrude slightly into the upper papillary dermis (Figure 1(c)). There is almost always an associated solar elastosis in the dermis, and a lack thereof can often be sufficient to prompt reconsideration of the diagnosis.

## 3. Squamous Cell Carcinoma *In Situ* (SCCS)/Bowen's Disease

Bowen's disease was first described by Bowen in 1912 [23], and is essentially equivalent to and used interchangeably with the term squamous cell carcinoma *in situ* (SCCS). Although it can present in individuals of any age, it is typically found in elderly patients, with a mean age of diagnosis exceeding 60 years [24]. Presentation in individuals under 30 years of age is extremely rare. 

Bowen's disease may arise on the skin of any body site: however, the vast majority of cases (approximately 72%) are found on sun-exposed surfaces such as the head, neck, and hands [25]. Mucosal surfaces and the nail bed are also commonly involved. Only in rare circumstances will SCCS be found on the palms of the hand or the soles of the feet [26]. Bowen's disease often presents as an asymptomatic, erythematous, well-demarcated, scaly patch or plaque. It tends to be slowly enlarging, and usually has a fairly irregular border. Lesions may become hyperkeratotic, crusted, fissured or ulcerated, and can occasionally be pigmented, especially when found in the genital region and the nails [26].

Bowen's disease can be considered a low-grade form of SCC, with the majority of studies reporting the risk of progression to SCCI at 3%–5%. The risk of invasive development is estimated to be slightly higher (approximately 10%) for genital Bowen's disease, also known as erythroplasia of Queyrat [27]. Despite the low incidence of malignant progression, Bowen's disease is of significant consequence since approximately 20% of the tumors that do develop into SCCI will eventually become metastatic [28]. The association of Bowen's disease with other forms of malignancy such as internal cancers is a highly controversial subject and continues to be an area of active research [29].

**Figure 2:**
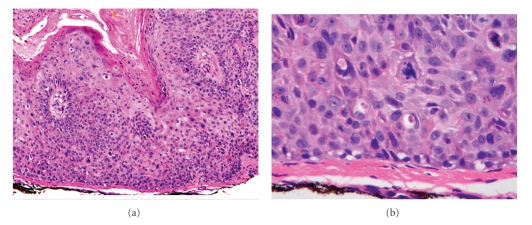
Squamous cell carcinoma in situ (SCCS)/Bowen's disease. (a) There is prominent dyskeratosis and aberrant mitoses at all levels of the epidermis, along with marked parakeratosis (100x). (b) Note, however, that the basement membrane remains intact (400x).

Histopathologically, the epidermis in Bowen's disease will show hyperkeratosis and parakeratosis. There will also be marked acanthosis with elongation and thickening of the rete ridges. These changes will overly keratinocytic cells which are often highly atypical and may in fact have a more unusual appearance than SCCI (Figure 2(a)). The atypia spans the full thickness of the epidermis, with the keratinocytes demonstrating intense mitotic activity, pleomorphism, and greatly enlarged nuclei. They will also show a loss of maturity and polarity, giving the epidermis a disordered or “windblown” appearance. Two types of multinucleated cells may be seen: the first will present as a multinucleated giant cell, and the second will appear as a dyskeratotic cell engulfed in the cytoplasm of a keratinocyte [25]. Occasionally, cells of the upper epidermis will undergo vacuolization, demonstrating an abundant and strongly eosinophilic cytoplasm. 

In contrast to AK's, the basal epidermal layer in Bowen's disease is frequently spared, and will show little to no visible atypia. Additionally, Bowen's disease will almost always involve both the interfollicular and adjacent follicular epithelium and adnexal structures [8]. The dermoepidermal junction will remain sharp and intact (Figure 2(b)), and there may be a mild to moderate lymphohistiocytic infiltrate detected in the upper dermis. 

## 4. Invasive Squamous Cell Carcinoma (SCCI)

The overwhelming majority of SCCIs (approximately 97%) are found in association with the malignant progression of an AK, and accordingly these two lesions are often thought of as different points along the same spectrum of disease [30]. SCCIs are often referred to as conventional SCCs.

**Figure 3:**
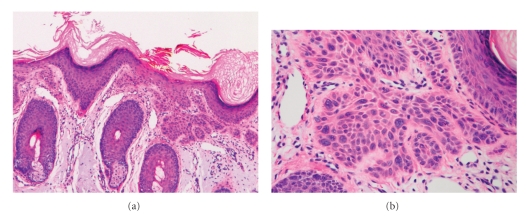
Superficially invasive squamous cell carcinoma (SCCSI). (a) These lesions often do not show the marked pleomorphism and atypical nuclei of in situ squamous cell carcinoma, but demonstrate early keratinocyte invasion of the dermis (150x). (b) Higher magnification demonstrates the pleomorphism of the invading keratinocytes (200x).

**Figure 4:**
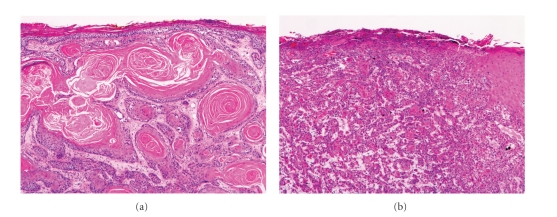
Invasive squamous cell carcinoma (SCCI). (a) Well-differentiated lesions show prominent keratinization and may form “pearl-like” structures where dermal nests of keratinocytes attempt to mature in a layered fashion (40x). (b) Moderately differentiated lesions of SCCI show much less organization and maturation with significantly less keratin formation (40x).

Histopathologically, SCCIs will frequently bear close resemblance to their precursor AK lesions, but can be distinguished from the latter via the presence of infiltrative cells passing through the basement membrane into the dermis (Figure 3) [6, 14]. This infiltrate can be somewhat difficult to detect in the early stages of invasion: however, additional indicators such as full thickness epidermal atypia and the involvement of hair follicles can be used to facilitate the diagnosis [31]. Later stages of invasion are characterized by the formation of nests of atypical tumor cells in the dermis (Figure 4), often with a corresponding inflammatory infiltrate. 

Additionally, SCCIs can be subdivided into three broad histologic grades based on their associated degree of nuclear atypia and keratinization. The majority of SCCI's arising from AKs will be well differentiated, with tumor cells containing only slightly enlarged, hyperchromatic nuclei with abundant amounts of cytoplasm. They will often produce large amounts of keratin, resulting in the formation of extracellular keratin pearls (Figure 4(a)). Intercellular bridges will frequently be visible. These tumors are generally associated with a very low-malignant potential, with the likelihood of metastasis being approximately 0.5% [12]. In contrast, SCCI can also present as a poorly differentiated tumor with greatly enlarged, pleomorphic nuclei demonstrating a high degree of atypia and frequent mitoses. Keratin production in these cells will be markedly reduced. This specific subtype of SCCI occurs much less commonly, and is typically associated with hypertrophic or proliferative AKs found on the ear and lip [32]. It will usually demonstrate a much more aggressive clinical behavior, with an increased rate of metastasis and recurrence [33]. A third, moderately differentiated subtype exists which will share features of both well-differentiated and poorly differentiated tumors (Figure 4(b)). 

## 5. Clear-Cell SCC

Clear-cell SCC is an extremely rare variant of SCC. It is commonly referred to as hydropic SCC due to the extensive hydropic degeneration of neoplastic cells, and the accumulation of intracellular fluid. It was first described by Kuo in 1980 [34], who reported six cases occurring in the head and neck region of elderly Caucasian males with histories of excessive sun exposure. Clinically, the lesions appear as nodules or ulcerated masses, and can easily be confused with sebaceous neoplasms, pilar tumors, and trichilemmal carcinomas.

**Figure 5:**
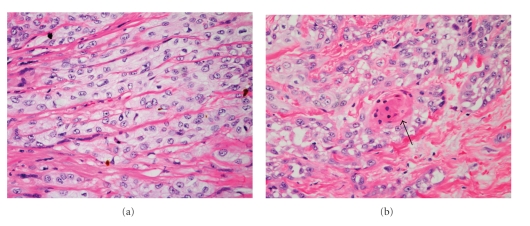
Clear-cell squamous cell carcinoma. (a) In this poorly differentiated example, attempts at keratinization are often no longer evident. The dysplastic cells here infiltrate in cords through the dermis (200x). (b) Unless other areas of the tumor show obvious squamous cell features such as seen here (arrow), immunostains will likely be required to classify this tumor (200x).

Histopathologically, Kuo [34] subdivided clear-cell SCC into 3 different categories: keratinizing (Type I), nonkeratinizing (Type II), and pleomorphic (Type III). Type I lesions are characterized as sheets or islands of tumor cells with clear, empty-appearing cytoplasm (Figure 5(a)). Cytoplasmic growth will displace cell nuclei to the periphery, causing these cells to be indistinguishable from regular mature adipocytes. Some cells will have a “bubbled” cytoplasmic appearance, leading them to resemble sebaceous neoplasms. The former can be distinguished from the latter through the additional presence of foci of keratinization and keratin pearls (Figure 5(b)). The surrounding stroma will be fibrotic, with a sparse inflammatory infiltrate. Type II lesions are predominantly dermal in origin, with no clear connection to the overlying epidermis. They are characterized by parallel or anastomosing cords of tumor cells separated by a compressed, fibrotic stroma with a dense inflammatory infiltrate. Tumor cells will have central nuclei with a finely reticulated clear cytoplasm, and evidence of central necrosis within tumor cords may be present. Ductal or glandular differentiation does not occur, and unlike Type I there is no keratinization. Type III lesions arise from the epidermis and show extensive ulceration. Atypical clear-cells display marked nuclear pleomorphism, with foci of squamous differentiation, areas of acantholysis, and the presence of dyskeratotic cells within pseudoglandular spaces. Perineural and vascular space will show considerable invasion. Of importance to note is that in all three types described above, there is no evidence of either glycogen or mucin found within the tumor cells, and only trace amounts of lipid. This is consistent with the hypothesis that clear-cell changes are degenerative [34]. 

Clear-cell SCC can resemble a variety of both benign and malignant entities such as clear-cell acanthoma, trichilemmoma, sebaceous neoplasms, and metastatic renal cell carcinoma. It can best be distinguished from these through the use of routine histology and immunohistochemical markers such as EMA, which will stain positively in sebaceous carcinoma [35], and metastatic renal cell carcinoma. Since it is found so rarely in the population, it is difficult to ascertain the malignant potential of this variant.

## 6. Spindle Cell (Sarcomatoid) SCC

Spindle cell SCC, also known as sarcomatoid SCC, is a rare variant of squamous cell that was first described by Martin and Stewart in 1935 [36]. It almost always occurs on areas of the skin with high levels of sun exposure, such as the head, neck, chest, and upper extremities, but can also occur in patients with histories of prior radiation exposure. While in the past this variant was thought to be primarily radiation induced, it is now known that it can arise *de novo* as well [36]. Those cases of spindle cell SCC that arise in sites of previous radiation tend to have a very aggressive course whereas those that are unrelated to radiation tend to be no more aggressive than conventional SCC. Clinically, spindle cell SCC will present predominantly in elderly Caucasian males as a raised or exophytic nodule, often with spontaneous bleeding and central ulceration. 

**Figure 6:**
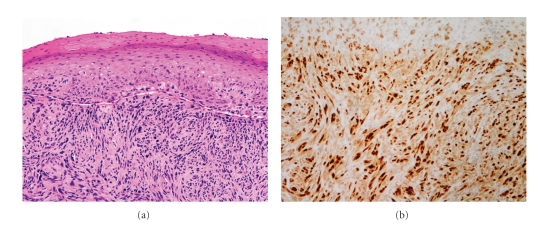
Spindle cell (sarcomatoid) squamous cell carcinoma. (a) The keratinocyte derivation of this lesion cannot be identified with certainty based solely on histology in this poorly differentiated tumor (100x). (b) Immunohistochemistry for p63 identifies the squamous origin of the tumor (100x).

Histopathologically, spindle cell SCC may be almost entirely composed of atypical spindle cells arranged in a whorled pattern (Figure 6(a)), or may have a combination of spindle cells and more conventional SCC cells, often with an associated AK [37]. Unlike conventional SCC, however, tumor cells will singly infiltrate the dermis without the formation of nests or cords. Connection to the overlying epidermis can vary. Bizarre pleomorphic giant cells, as well as heterologous elements with numerous mitotic figures can be identified, often with deep infiltration into the dermis, subcutis, fascia, muscle, and even occasionally bone. The stroma, however, should not be significantly desmoplastic. 

In the absence of keratin pearls and connection to the epidermis, spindle cell SCC can be difficult to distinguish from conditions such as atypical fibroxanthoma, spindle cell melanoma, or spindle cell sarcoma. In these cases, the use of immunohistochemical (IHC) stains can prove extremely useful for the diagnosis. Spindle cell SCC will stain positively for high molecular-weight cytokeratins such as CK 5/6, as well for EMA antibodies. It will also variably stain for vimentin [9]. Recently p63, a member of the p53 gene family expressed in the nuclei of basal and spinous cells of the epidermis, has been found to be a useful nuclear marker in the differentiation of spindle cell SCC from other histologically similar conditions (Figure 6(a)) [38].

## 7. SCC with Single Cell Infiltrates

A relatively rare variant of SCC is one which displays single cell infiltrates. This variant is generally found on the face and neck of older individuals, and is thought to be more aggressive on average than conventional SCC [39]. This may, in part, be due to the singular nature of the cellular atypia, which can allow for the lesions to frequently go unnoticed or misdiagnosed. The lack of an appropriate and timely response may lead to a higher rate of metastasis and recurrence. 

Histopathologically, single-cell SCC is composed almost entirely of singular atypical cells, which exist either individually or loosely arranged as nests in the dermis. There is an overall lack of cohesiveness amongst atypical cells, and usually no connection to the overlying epidermis or adnexal structures. Often they are found in regions displaying large amounts of solar elastosis. This variant is incredibly difficult to diagnose since single cells can often be obscured by an adjacent inflammatory infiltrate, and can occasionally resemble spindle cell melanoma or atypical fibroxanthomas [39]. The most effective means of diagnosing this rare variant is through the use of immunohistochemical staining, in particular, the p63 nuclear maker, as well as the cytokeratin antibody stain MNF116 [39]. Specifically, p63 will help indicate the degree of epithelial differentiation [38], while MNF116 has been shown to stain cytokeratin expressed strictly in cutaneous tumors as opposed to mesenchymal or melanocytic lesions [40]. Both these stains also have high sensitivities, allowing for effective staining of even poorly differentiated tumors.

## 8. *De Novo* SCC

*De novo* SCC is a particularly aggressive variant of SCC which lacks a precursor and will thus arise independently on skin which has been chronically injured or diseased. It is not correlated with sun exposure, nor will it show any evidence of previous actinic lesions or SCCS. Generally, *de novo* SCC will emerge in the setting of long-standing ulcers, burn scars, or osteomyelitis. It can also be seen in chronic inflammatory conditions such as discoid lupus erythematosus and dystrophic epidermolysis bullosa [41–44]. The tendency for malignancies to develop in burn scars was first described by Marjolin in 1827, and accordingly, are referred to today as Marjolin's ulcers [45]. 

Unlike conventional SCCs, which tend to occur on the face, neck, hands, and other sun-damage prone areas, *de novo* SCCs are most commonly found on the lower extremities, where burn scars are more prevalent. Although females on average sustain a greater number of burn injuries than males, there is a predilection for malignant scar development in men, and *de novo* SCCs are significantly more likely to be found in males than in females [46]. They usually emerge 20 to 40 years after the original trauma occurred, and will present clinically as nonhealing, exophytic growths or indurated ulcers which have recently became painful and may have a foul smelling discharge [46].

**Figure 7:**
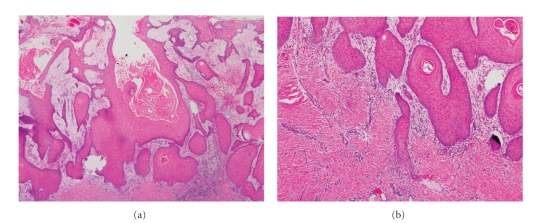
Squamous cell carcinoma *de novo* arising in a Marjolin's ulcer. (a) These tumor are often well differentiated as seen here (20x). (b) Note the dense scarring at the base of the tumor (40x).

Histopathologically, *de novo* SCCs will resemble fairly well-differentiated conventional SCCs, however, they will be seen with an associated ulcer or scar rather than an actinic keratosis (Figure 7). Additionally, they can be distinguished from conventional SCCs via the absence of solar elastosis in the dermis. Centrally, the lesion may be atrophic or ulcerated, with invading strands of tumor cells appearing from the epidermis or ulcer edge. These lesions are extremely aggressive, with the likelihood of regional lymph node metastasis reaching as high as 54% [46]. The incidence of recurrence is also dramatically increased, and the overall prognosis is poor, with a 5-year survival rate of only 52%–75% [8]. Lesions of the lower extremities generally have a greater metastatic potential and higher incidence of recurrence than those found in other anatomic locations such as the face and trunk [46].

## 9. Verrucous Carcinomas

Verrucous carcinoma (VC) is thought to be a relatively indolent form of SCC that presents with bland, verrucous-like features. It was first described by Ackerman in 1948, and is associated with both low-risk (types 6 and 11) and high-risk (types 16 and 18) types of HPV [47]. Although Ackerman's report described a low-grade, well-differentiated tumor seen in the oral cavity [48], VC has subsequently been seen on several different sites of the body, and is currently subdivided into 4 main clinicopathologic categories based on the associated area of involvement [49]. These include oroaerodigestive VC (also known as Ackerman tumor or oral florid papillomatosis), anourogenital VC (also known as Buschke-Lowenstein tumor), palmoplantar VC (also known as epithelioma cuniculatum), and VC found on other cutaneous sites. 

Oroaerodigestive VC is thought to be the most common form of VC and accounts for anywhere between 2%–12% of all oral carcinomas [50]. It is generally seen in elderly Caucasian males on the gingival and buccal mucosa, but has been known to involve the larynx as well. Early lesions will appear as white, translucent, keratotic patches found on an erythematous base, and will subsequently develop into soft, rubbery papillary growths with pebbly surfaces. Ulceration and fistulation are also commonly seen, with occasional local invasion into soft tissue and bone. Oroaerodigestive VC has strong positive associations with both low- and high-risk HPV, with high-risk HPV presenting in up to 45% of laryngeal VC cases [51]. It has also been linked to chemical carcinogens such as chewing tobacco and snuff [52], as well as other conditions affecting the oral mucosa such as lichen planus, chronic candidiasis, leukoplakia, and chronic lupus erythematosus [53]. 

Anourogenital VC is thought to account for approximately 5%–24% of all penile carcinomas [54], and typically presents on the glans penis and prepuce of middle-aged uncircumcised males. It is only rarely found in the female genital tract. Lesions will appear large, exophytic, and cauliflower-like, with verrucous or ulcerated surfaces. This variant of VC is most commonly associated with low-risk HPV, with a positive correlation found in up to 50% of cases, but it can be seen in association with high-risk types as well [54]. 

Palmoplantar VC is generally found on the soles of elderly Caucasian males, but may also be seen on the toes, the heel, or the dorsum of the foot [55]. Lesions are often initially mistaken for plantar warts, however, they tend to slowly evolve into bulky exophytic masses with ulceration and foul-smelling discharge. It is thought to be associated loosely with the low-risk HPV types, but is also related to trauma, chronic irritation, and other forms of HPV infection [49]. 

VC lesions found on other cutaneous sites are rare occurrences, and tend to appear as slow-growing warty or cauliflower-like lesions with only weak associations to HPV infections [49]. 

**Figure 8:**
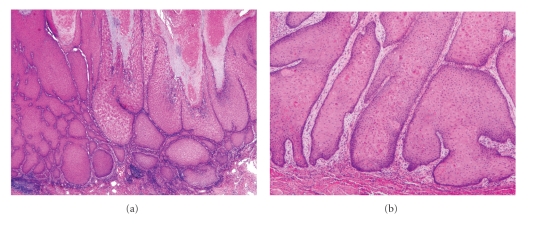
Verrucous carcinoma. (a) Scanning magnification demonstrates massive acanthosis and parakeratosis (40x). Higher magnification highlights the broad pushing borders (100x).

Despite these separate subclassifications, all four forms of VC share a similar histopathological description. The epithelium will generally display a characteristic endoexophytic growth pattern, with a prominent granular layer showing marked hyperkeratosis and parakeratosis, as well as acanthosis and papillomatosis (Figure 8(a)). Keratinocytes are often enlarged with prominent nuclei, but atypia is otherwise minimal. The epidermis remains well differentiated with obvious stratification, and will demonstrate broad, bulbous, rete-like projections which will descend deep into the underlying dermis (Figure 8(b)). These will generally be surrounded by a dense inflammatory infiltrate, and can often result in the formation of sinuses and keratin-filled cysts [47]. The projections have a remarkably indolent appearance, and lack all the classical signs of invasion such as cellular atypia and infiltration. Malignant changes are therefore the result of compressive destruction rather than invasion, and the margins of VC lesions will often show aggressive borders which may extend into the adjacent structures causing destruction of the local connective tissue, muscle, cartilage, or bone [49]. 

Additionally, reports of “hybrid” tumors have been described where lesions present with the characteristic features of VC described above, but will also show focal areas of invasive tumor cells which will behave in a similar manner to conventional SCC [56, 57]. Arriving at the proper diagnosis of SCC versus VC becomes clinically relevant since SCC has a much higher likelihood of lymph node metastasis, and usually requires an alternate treatment plan. The use of immunohistochemical stains, specifically bcl-2, Ki-67, and p53, are instrumental in this process. Only the basal proliferating cells in the lower third of the epidermis will stain in VC whereas the entire epidermis will stain positively in SCC [58]. Based on the relative degree of staining, one can distinguish between the two.

## 10. Lymphoepithelioma-Like Carcinoma of the Skin

Primary LELCS is a rare variant of SCC which was first described by Swanson et al. in 1988 [59]. Lesions occur on the head and neck of elderly patients, with no significant sex predilection. They often present as slowly growing dermal nodules, and will only rarely show signs of ulceration. 

**Figure 9:**
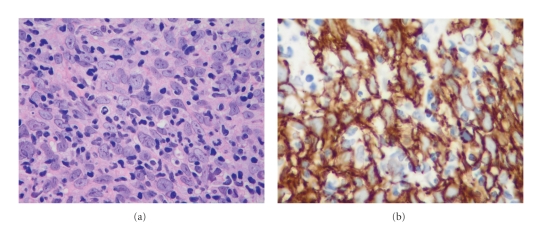
Lymphoepithelioma-like carcinoma. (a) This tumor is composed of large epithelioid cells with a marked lymphocytic infiltrate (400x). (b) Immunohistochemistry for pancytokeratin demonstrates that the tumor cells are keratinocytes (600x).

Histopathologically, islands or syncytial sheets of tumor will be seen in the mid-to-deep dermis. These are composed of large, pale, eosinophilic polyhedral cells, with vesicular nuclei and prominent nucleoli (Figure 9(a)). The cells tend to be cohesive and atypical, exhibiting numerous mitoses and lacking any distinct cellular borders. Additionally, the presence of an immune infiltrate will be visible surrounding the atypical aggregates, although the degree to which infiltration occurs has been known to show marked variation. For instance, while the immune response can be sparse and peripherally located, it can also be extremely dense, obscuring the nearby tumor cells. This infiltrate is composed largely of plasma cells and small lymphocytes, however the occasional neutrophil and eosinophil can also be found [59]. 

Even though squamous differentiation is not always apparent in LELCS, it is still considered a form of SCC since cells will show desmosomes and tonofilaments under electron microscopy [60]. Additionally, adnexal and trichilemmal differentiation is commonly seen, and there are several reports in which SCC *in situ* has been found in the overlying epidermis [61]. LELCS can often be quite difficult to identify microscopically, due in large part to the morphologic similarities between atypical keratinocytes and the surrounding lymphocytic infiltrate. The use of immunohistochemical staining, in particular the cytokeratin stain AE1/AE3 (Figure 9(b)) and the epithelial membrane antigen (EMA) stain, can be essential for correct diagnosis [62].

## 11. Additional Prognostic Factors

The unique histopathological features seen in a given SCC lesion are tremendously important in predicting its malignant potential. There are several other critical features, however, which may have equal if not greater prognostic value and must be assessed when evaluating tumor risk. These include tumor size and depth of invasion, degree of differentiation, anatomical location, perineural, and perivascular invasion, and immunosuppression [33]. These are not necessarily mutually independent variables in that certain histologic subtypes of SCC are strongly associated with a specific set of secondary prognostic features. The primary histological differences between low-grade and high-grade SCC are summarized in Table 1.

Size and depth of invasion are perhaps the most important determinants of the likelihood of tumor recurrence and metastasis [33]. As a broad rule, tumors less than 2 cm in size will only rarely metastasize and are unlikely to recur whereas those greater than 2 cm in size pose a significant threat of metastasis and recurrence [63]. Similarly, tumors that exceed 4 mm in depth or Clark's level III, showing involvement of both the deeper levels of the dermis as well as the subcutaneous tissue, have a much more aggressive course of action and over a seven-fold increase in the probability of metastasis [64, 65]. Skin lesions greater than 8 mm in depth or Clark's level V pose such a significant threat of metastasis that nodal involvement and prophylactic node dissection should be seriously considered [65]. As expected, SCCI, which is derived largely from a superficial precursor lesion, is considered to be an indolent tumor. In contrast, SCC with single cell infiltrates, which is defined predominantly by dermal activity, is considered to be a more aggressive tumor type. 

The degree of histologic differentiation, as well as the anatomic site of the lesion, will also play a role in SCC evaluation and prognosis. Poorly differentiated tumors, particularly from the ear or the lip, will be three times more likely to metastasize, and twice as likely to recur when compared to tumors that are well differentiated [33]. Despite this, the majority of metastatic tumors will be moderately to well differentiated, highlighting the importance of assessing all the prognostic factors in the evaluation of metastatic potential [33]. 

Another essential component in assessing the malignant potential of a tumor is the presence of perineural and perivascular spread. Perineural involvement (PNI) is thought to occur in approximately 14% of all SCC tumors arising on the head or neck [66], and is indicative of the inherently aggressive nature of the tumor. Accordingly, tumors with PNI will show a much greater likelihood of local recurrence (23%) relative to those without (9%) [67]. They will also be associated with a worse overall outcome, and a significant increase in the disease-specific mortality rate. Clinically, perineural involvement can present as conventional SCC with an associated numbness, facial muscle weakness, twitching, or visual change. However, there are often no clinical symptoms of nerve involvement, and PNI is most frequently diagnosed microscopically [66, 67].

Similarly, invasion of capillary lymphatics signifies a more aggressive tumor nature and is correlated with an increased incidence of metastases, local recurrence, and disease-specific death [67]. Additionally, SCC metastasis occurs predominantly via local lymphatics and often deposits in the lymph nodes of the neck [68]. Although invasion tends to remain localized to regional nodes, prognosis remains extremely poor with only a 34.4% cure rate [33]. 

**Figure 10:**
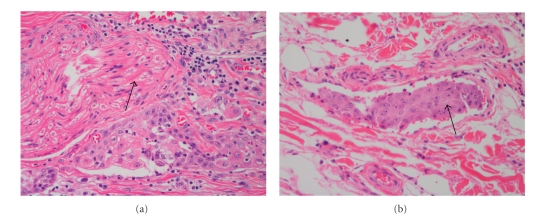
Poor prognostic factors. (a) Perineural invasion: the arrow indicates a large peripheral nerve that has been surrounded by tumor cells (200x). (b) Vascular invasion: the arrow indicates a small cluster of atypical squamous cells in a small vessel (200x).

Histopathologically, PNI and perivascular invasion will appear as an overlying SCC with atypical tumor cells which have penetrated the nerve or vascular tissue (Figure 10). This can present in a variety of different invasion patterns, most frequently involving a complete encircling of the nerve or vessel by tumor cells. An incomplete, crescent-like pattern of atypical cells is also commonly seen. Occasionally, tangential contact, permeation, and lamination can be observed. Invasion almost always occurs contiguous to the main body of the tumor; however, it has been known on occasion to affect more distant nerve and vascular sites. Usually, tumor cells arranged in solid or sheet-like patterns are less invasive, and will pass around the nerve or vessel. In contrast, individual tumor cells will generally penetrate and track along associated structures [66, 67]. 

 Finally, host immunosuppression can greatly increase the likelihood of SCC development, recurrence, and malignant spread [69–72]. Suppression may be due to an underlying malignancy, the active use of immunosuppresive agents during transplant therapy, or infection with HIV. In fact, NMSC is considered to be one of the most common side effects of long-term immunosuppressant use seen in transplant recipients. Unlike the general population, these patients are more likely to present with SCCs than BCCs [73, 74]. These lesions will typically emerge on the sun-exposed surfaces of the body, and will be found more frequently in patients with histories of sun exposure [75] and HPV infection [76]. Immunosuppressed patients will usually present with multiple SCC lesions, and while the risk of each individual lesion metastasizing is not markedly elevated, the presence of so many will have the effect of increasing the overall risk of metastasis [77, 78]. 

Several methods of treatment exist for SCCs, most of which have been shown to be extremely effective in the management of these lesions. These include cryotherapy, curettage, electrodesiccation, radiation, surgical excision, and Mohs micrographic surgery [33]. While most non-Mohs modalities have equal cure rates for low-risk, indolent SCC's, they have relatively poor outcomes when dealing with more aggressive tumors [33]. Mohs micrographic surgery remains the treatment of choice for SCC lesions associated with any high-risk prognostic factor. When this therapy is not suitable for use, as is often the case with tumors located on the face, radiation and chemotherapy prove to be viable alternatives.

## 12. Conclusions

SCC's are often considered to be a single class of lesions associated with relatively benign outcomes and a low risk of metastasis. However, these lesions can show dramatic histopathological diversity and are associated with a wide diversity of clinical outcomes. The ability to identify SCC variants with divergent clinical behaviors is of great importance in the assessment of tumor risk. To this end, we have provided a detailed and descriptive outline for the histological distinction of the most commonly encountered SCC lesions, highlighting those variants which will be associated with more aggressive behaviors and a worse clinical prognosis. The majority of SCC lesions will begin as noninvasive precancerous neoplasms, which arise as a direct result of excessive sun exposure. SCC precursors include both AKs and SCC* in situ* (Bowen's disease). These lesions will progress and evolve to give rise to SCCI, as well as several rare subtypes including clear-cell SCC, spindle cell SCC, and SCC with single cell infiltrates. Additionally variations of SCC which are unrelated to solar exposure include* de novo* SCCs, VCs, and LELCS. In particular, SCC with single cell infiltrates as well as *de novo* SCC will show more aggressive patterns of behavior. Additionally, large tumors which are poorly differentiated and show deep infiltration into the dermis and subcutaneous tissues will be associated with a higher likelihood of recurrence and metastases. Understanding how to differentiate between these variants of SCC microscopically, with the additional benefit of immunohistochemical staining, will enable a more informed and timely selection of treatment options, ensuring the best possible results for the patient.

##  Conflicts of Interest

The authors have no conflicts of interest to declare.

## Figures and Tables

**Table 1:** Histological differences between low-grade and high-grade SCC.

	*Low-Grade SCC*
	Well to moderately differentiated: intercellular bridges and keratin pearls
	Tumor cells arranged in solid or sheet-like patterns
	Association with solar damage and precursor actinic keratosis
	Diameter less than 2 cm
	Depth less than 2 mm

	*High-Grade SCC*
	Poorly differentiated: clear-cell, sarcomatoid, or single cell features
	Presence of infiltrating individual tumor cells
	Arising *de novo* or in site of prior injury (ulcer, burn scar, or osteomyleitis)
	Perineural and/or perivascular invasion
	Diameter greater than 2 cm
	Depth greater than 2 mm
